# Social Perception, Trust, and Reluctance Towards Vaccines: A Bibliometric Analysis (2019–2025)

**DOI:** 10.3390/ijerph23010119

**Published:** 2026-01-18

**Authors:** Johanna Valeria Caranqui-Encalada, Grecia Elizabeth Encalada-Campos, Joceline Damaris Caranqui-Encalada, Carmen Azucena Yancha-Moreta, Dennis Alfredo Peralta-Gamboa

**Affiliations:** 1 Facultad de Salud y Servicios Sociales, Universidad Estatal de Milagro, Milagro 091050, Ecuador; jcaranquie1@unemi.edu.ec (J.V.C.-E.); cyancham@unemi.edu.ec (C.A.Y.-M.); 2 Hospital General IEES Milagro, Milagro 091050, Ecuador; joceline.caranqui@iess.gob.ec; 3 Acompañamiento Estudiantil, Escuela de Salud, Facultad de Posgrados, Universidad Estatal de Milagro, Milagro 091050, Ecuador

**Keywords:** vaccine hesitancy, disinformation, health communication, social media, vaccination behavior

## Abstract

The objective of this study was to analyze social perception, trust, and vaccine hesitancy through a combined approach of bibliometric analysis and qualitative synthesis, based on the most cited articles in the recent scientific literature. A systematic search was conducted in indexed databases, identifying patterns of production, collaboration, citation, thematic networks, and conceptual trends associated with the study of public trust in vaccines. The results reveal a marked geographic concentration of scientific production, dominated by the United States and the United Kingdom, as well as a strong articulation of thematic clusters linked to digital disinformation, health communication, risk perception, and psychosocial determinants of vaccine acceptance. The qualitative synthesis of the most influential studies reveals that vaccine hesitancy is a multidimensional phenomenon, determined by sociocultural, cognitive, emotional, and structural factors that interact dynamically according to each context. Disinformation, institutional trust, community narratives, and the credibility of sources emerge as central components in individual decision-making. Together, the integrated results enable a deeper understanding of vaccine hesitancy beyond traditional cognitive models, highlighting the need for contextualized communication strategies, intercultural approaches, and health policies based on trust and social participation. This study provides an integral view of the scientific landscape and establishes priority lines for future research and the design of effective public health interventions.

## 1. Introduction

Vaccine hesitancy has become one of the main threats to contemporary public health, being particularly intensified during the COVID-19 pandemic. This phenomenon, defined as the delay or refusal to vaccinate despite its availability, cannot be explained solely as an information deficit, but as a complex process traversed by sociocognitive, emotional, and sociocultural factors [1,2]. Too, Vaccine hesitancy—defined by the World Health Organization’s Strategic Advisory Group of Experts (SAGE) on Immunization as the delay in acceptance or refusal of vaccination despite the availability of vaccination services—is a complex and context-specific phenomenon [3]. The pandemic profoundly modified information ecosystems and amplified the circulation of rumors, conspiracy narratives, and disinformation, which generated uncertainty and eroded institutional trust even in populations previously favorable to vaccination [4,5].

In recent years, the scientific literature on social perception, trust, and vaccine reluctance has grown rapidly, although with a marked geographic and epistemological concentration. Bibliometric evidence shows that the United States and the United Kingdom lead global production and citation, configuring a centralized knowledge structure [6,7]. This predominance of the Global North conditions theoretical and methodological perspectives, while regions such as Latin America, Africa, or the Middle East—where the determinants of hesitancy differ substantially—remain underrepresented despite the significant sociocultural and structural challenges they face [8,9].

At the thematic level, the most cited studies agree insofar as pointing out that vaccine hesitancy emerges from the interaction between institutional trust, risk perception, collective emotions, exposure to contradictory information, and previous experiences with health systems. For example, research such as that of Dempsey et al. [10] and Middleman et al. [11] highlights that the recommendation from healthcare personnel is the most robust predictor of vaccine acceptance. On the other hand, studies focused on disinformation, such as those by Wawrzuta et al. [12] and Bradshaw et al. [5], show that anti-vaccine narratives have a greater emotional load and penetration in social networks, shaping attitudes even in individuals with a good educational level.

Likewise, psychosocial determinants—including anxiety, fear of contagion, perceptions of vulnerability, or emotional saturation—play a decisive role in crisis contexts [13,14]. Research from regions with strong cultural or religious influence shows that vaccine hesitancy cannot be detached from community identity, social hierarchies, and local trust narratives [15,16].

However, although the volume of scientific production has increased significantly, methodological and geographic gaps persist that limit comprehensive understanding of the phenomenon. Most studies are cross-sectional, focused on urban and highly digitized contexts, which makes it difficult to capture the temporal transformations of hesitancy or its expression in vulnerable, rural populations or those with low health literacy [17,18].

In this context, the present study aims to analyze, in an integrated manner, social perception, trust, and vaccine hesitancy through a combined approach of bibliometric analysis and qualitative synthesis of the most cited articles in the field. This approach allows not only mapping the global structure of knowledge, but also understanding the meanings, patterns, and determinants underlying vaccine hesitancy in diverse contexts, providing critical evidence for the design of public policies, communication interventions, and future research agendas.

## 2. Methodology

The present study adopts a bibliometric and qualitative design of a descriptive and exploratory nature, with the purpose of analyzing the scientific evolution, conceptual approaches, and thematic trends on social perception, trust, and reluctance toward vaccines between the years 2000 and 2025.

The approach combines quantitative methods—to examine patterns of productivity, impact, and scientific collaboration networks—with a qualitative component oriented toward the interpretation of discourses and social dimensions associated with the acceptance or rejection of vaccines in the region.

### 2.1. Sources of Information and Search Strategy

The collection of documents was carried out in the Scopus and Web of Science (WoS) databases, selected for their multidisciplinary coverage in medical sciences, social sciences, and public health. In WoS, the Social Sciences Citation Index (SSCI) and Science Citation Index Expanded (SCI-EXPANDED) collections were used, which ensure representativeness of studies on social trust, health, and public communication.

The search strategy was designed using Boolean operators to combine two main conceptual blocks aligned with the objectives of this study. The first block captured vaccine-related phenomena, including hesitancy, confidence, refusal, acceptance, and uptake. The second block focused on social, perceptual, and communicational dimensions, such as social perception, trust, health communication, and misinformation. Within each block, synonymous and related terms were combined using the OR operator, while the two conceptual blocks were connected using the AND operator. Searches were conducted in the title, abstract, and keywords fields (TITLE-ABS-KEY in Scopus and TS in Web of Science), using terms in English, Spanish, and Portuguese.

Scopus: TITLE-ABS-KEY ((“vaccine hesitancy” OR “vaccine reluctance” OR “vaccine refusal” OR “vaccine confidence” OR “trust in vaccines” OR “vaccine accept” OR “vaccine uptake” OR “reticencia a las vacunas” OR “aceptación de la vacuna” OR “hesitação vacinal”) AND (“social perception” OR “public perception” OR attitudes OR opinions OR beliefs OR “social trust” OR “health communication” OR misinformation OR disinformation OR “fake news” OR “social media” OR “redes sociales”)).

Web of Science (WoS): TS = ((“vaccine hesitancy” OR “vaccine reluctance” OR “vaccine refusal” OR “vaccine confidence” OR “trust in vaccines” OR “vaccine accept” OR “vaccine uptake” OR “reticencia a las vacunas” OR “aceptación de la vacuna” OR “hesitação vacinal”) AND (“social perception” OR “public perception” OR attitudes OR opinions OR beliefs OR “social trust” OR “health communication” OR misinformation OR disinformation OR “fake news” OR “social media” OR “redes sociales”)).

The following filters were applied from the Scopus and WoS platforms: Period: The temporal range was defined between 2019 and 2025, considering the surge in debates on vaccine trust, disinformation, and social perception, especially after the COVID-19 pandemic. Document Type: Only original articles and systematic reviews published in indexed scientific journals and peer-reviewed were included. Language: Records in English, Spanish, or Portuguese.

### 2.2. Inclusion and Exclusion Criteria

Inclusion Criteria:

- Original articles and reviews that analyze perceptions, attitudes, trust, or reluctance toward one or more vaccines.
- Studies with populations or Latin American contexts.
- Documents that include quantitative or qualitative methodologies linked to trust, disinformation, or vaccination behavior.
- Peer-reviewed publications with verifiable methodological information.

Exclusion Criteria:

- Purely clinical or epidemiological studies without analysis of perception or trust.
- Duplicate documents or those with restricted access without a verifiable abstract.
- Non-indexed literature, editorials, or institutional reports without peer review.

### 2.3. Data Extraction and Cleaning Procedure

The records obtained from Scopus and WoS were exported in .csv and .xlsx formats, respectively, and then integrated into a consolidated database using R software (version 4.4.3).

The processing stages were as follows:

1. Standardization of metadata: conversion of titles to lowercase and cleaning of special characters.
2. Removal of duplicates: using the duplicated() functions and matches via grepl() in R.
3. Thematic filtering: selection of articles with the presence of terms such as vaccine hesitancy, vaccine confidence, social perception, or equivalents in the title/abstract. This process was carried out with the help of the grepl() library to search for exact matches with the previously described search terms.
4. Manual validation: review of titles and abstracts to confirm thematic relevance and alignment with the study objectives. This step ensured that each article explicitly addressed issues related to vaccine hesitancy, trust, social perception, health communication, or misinformation in the context of vaccination. Articles focusing exclusively on clinical, immunological, or epidemiological aspects without a social or perceptual component were excluded. This manual validation was not intended to apply geographic filters, but to ensure conceptual consistency and analytical relevance within the global scope of the bibliometric analysis.

### 2.4. Bibliometric Analysis

The initial search yielded 11,951 documents in Scopus and 6675 documents in WoS. After applying the document type and language filters, 8474 articles were downloaded from Scopus and 4999 from WoS. A total of 317 duplicate records were removed; after reviewing titles and abstracts, 900 documents remained. Finally, 826 documents were selected after reading the full articles.

The bibliometric analysis was executed with R (v4.4.3) and VOSviewer (v1.6.20) to map the scientific structure of the field. The following packages were used:

- readxl, data.table → import and management of records.
- dplyr, tidyverse → data cleaning and filtering.
- ggplot2 and gridExtra → visualization of publication and citation trends.
- openxlsx → export of results.

The indicators analyzed were as follows:

- Annual productivity (number of articles per year).
- Scientific impact (accumulated and average citations per document).
- Most influential journals and authors.
- Leading countries and international collaboration networks (VOSviewer).
- Keyword co-occurrence map, to identify thematic clusters on trust, disinformation, social perception, vaccination policies, and digital media.

### 2.5. Qualitative Review

From the total articles included, those with more than 30 citations were selected for further qualitative examination. This subset was chosen to represent the most influential studies in the field, based on citation impact and relevance to the study objectives. All articles included in the bibliometric dataset met the predefined inclusion criteria and were peer-reviewed publications. The qualitative phase consisted of a structured content analysis focused on communicational, sociocultural, and institutional dimensions, rather than a fully inductive qualitative coding process. In total, *n* = 48 articles were included in this qualitative content analysis.

The citation threshold (*n* > 30) was applied as a pragmatic criterion to identify highly influential studies within the global literature, allowing for a focused qualitative content synthesis of publications that have substantially shaped academic debates on vaccine hesitancy. This threshold was not intended to ensure geographic representativeness, but rather to capture conceptual and thematic influence. Consequently, this approach may privilege globally visible research, often originating from Global North contexts. This limitation is acknowledged and reflects the objective of analyzing dominant narratives and frameworks in the international literature, rather than providing a region-specific qualitative representation.

The examination focused on three analytical dimensions:

1. Communicational dimension: mechanisms of information, disinformation, and the role of traditional and digital media in shaping social perceptions about vaccines.
2. Sociocultural dimension: religious, political, and community factors that influence trust or reluctance toward vaccination.
3. Institutional dimension: the role of public policies, health systems, and trust in health authorities.

This analytical framework guided the qualitative content synthesis, allowing the identification of recurrent patterns, gaps, and dominant approaches across the reviewed studies. Specifically, insights derived from the communicational, sociocultural, and institutional dimensions were systematically integrated to construct the subsequent synthesis and typology of research on vaccine trust and hesitancy. This process resulted in the identification of dominant thematic orientations in the literature, including public perception, media coverage, vaccination campaigns, the effects of digital disinformation, and trust in health institutions.

This analysis allowed for the identification of patterns, gaps, and dominant approaches, as well as the formulation of a typology of studies on trust and vaccine reluctance in Latin America (for example, public perception, media coverage, vaccination campaigns, effects of digital disinformation, and trust in health institutions).

## 3. Results

### 3.1. Analysis of Productivity and Citations

**Figure 1:**
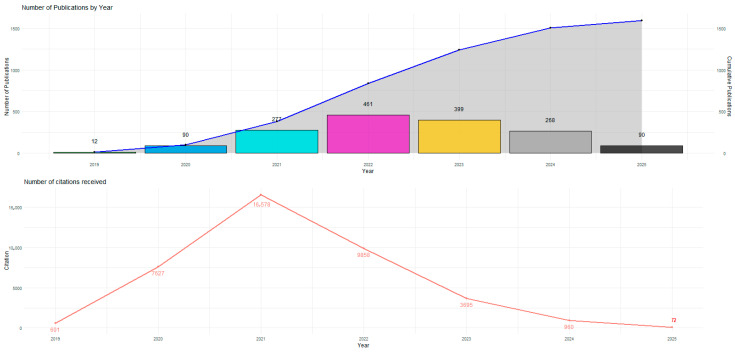
Temporal Evolution of Scientific Productivity and Citation Impact in Studies on Social Perception, Trust, and Vaccine Hesitancy.

Figure 1 reveals a clearly segmented dynamic in the scientific production on social perception, trust, and reluctance toward vaccines in Latin American contexts, reflecting the structural influence of the COVID-19 pandemic on global research agendas. The observed behavior is articulated in three analytically differentiable phases, which allow for the interpretation of the field’s consolidation, early maturity, and subsequent deceleration.

Exponential Growth and Critical Inflection (2019–2021)

The initial period shows an abrupt increase in both productivity and the impact of publications. Between 2019 and 2020, the number of articles rose from 12 to 90, representing an increase of more than 650%. This growth intensified in 2021, with 277 publications recorded, making that year the point of greatest expansion in the field.

Parallel to this, citations experienced an even more pronounced rise from 601 in 2019 to 7627 in 2020, reaching a historical maximum in 2021 with 16,578 citations. This pattern confirms that the health emergency acted as an unprecedented catalyst, directing scientific efforts toward understanding attitudes, perceptions, institutional trust frameworks, and disinformation processes linked to mass vaccination campaigns.

Thus, 2021 constitutes an epistemic and bibliometric inflection point, in which the field not only expands its production volume but also gains centrality in interdisciplinary debates connecting public health, digital communication, social sciences, and behavioral psychology.

Reconfiguration and Progressive Decline (2022–2024)

Although 2022 recorded 461 documents—the second highest value for the period—this increase should be interpreted as a carryover effect from research developed during the health crisis, much of which was published in a deferred manner. However, citations decreased to 9858, marking the beginning of a contraction in the field’s visibility and impact.

From 2023 onward, the decline became sustained: production fell to 399 articles, and citations reduced significantly (3695), evidencing a loss of thematic centrality once the most critical phase of the pandemic was overcome. In 2024, this trend intensified with a decrease to 268 publications and only 960 citations, reflecting a gradual return to levels comparable to those observed before the pandemic period.

This phase can be characterized as a process of structural deceleration, in which topics related to vaccine hesitancy compete with new research priorities in public health and post-COVID social studies.

Post-Crisis Stabilization and Return to Scientific Normalcy (2025)

The reduction to 90 publications and 72 citations in 2025 indicates a stabilization of the field at levels similar to the pre-pandemic period. This normalization suggests that scientific interest is no longer concentrated on the health emergency, but rather on broader debates about institutional trust, health governance, risk communication, and disinformation in digital environments.

Far from representing a thematic decline, this stabilization indicates that the field has transitioned from a phase of hyper-reactive growth to a stage of analytical maturation, where studies tend to focus on comparative frameworks, sociocultural approaches, and longitudinal evaluations.

Critical Interpretation of Bibliometric Behavior

The analysis of Figure 1 allows us to conclude the following:

- The evolution of the field is closely associated with global crisis dynamics, which explains the concentration of publications and citations between 2020 and 2021.
- The subsequent decline does not imply a loss of relevance, but rather a redistribution of scientific interest toward new questions related to informational resilience, citizen trust, and long-term vaccination policies.
- The observed temporal structure is consistent with bibliometric patterns characteristic of global disruptive events, where scientific production is organized into an expansive phase, a contraction phase, and a stabilization stage.

### 3.2. Journals with Greatest Productivity and Impact

Table 1 presents the journals that concentrate the highest scientific production and citations related to social perception, trust, and reluctance toward vaccines during the analyzed period. The results evidence a highly specialized editorial core in vaccines, public health, and digital communication, confirming the multidisciplinary nature of the field.

Predominance of Specialized Vaccine Journals

The top positions are occupied by journals directly linked to immunology, vaccinology, and the evaluation of vaccination policies:

- Vaccines (135 articles; 3969 citations).
- Vaccine (103 articles; 2236 citations).
- Human Vaccines and Immunotherapeutics (57 articles; 2742 citations)

This group concentrates more than 45% of all identified production, evidencing that research on vaccine perception and trust is predominantly published in journals with an explicit focus on biomedicine and vaccinology. Additionally, these journals show high citation levels, indicating that the studies published in them have substantial impact within the international scientific community.

Relevance of Public Health and Epidemiology Journals

A second group, integrated by public health journals, amplifies the interdisciplinary perspective of the field:

- BMC Public Health (40 articles; 1092 citations).
- Frontiers in Public Health (40 articles; 570 citations).
- International Journal of Environmental Research and Public Health (37 articles; 1266 citations).
- BMJ Open (26 articles; 665 citations)

These journals address, from epidemiological and social perspectives, topics such as vaccine reluctance, health inequities, preventive behaviors, health literacy, and sociocultural factors that modulate vaccine acceptance. The sustained presence of articles in these sources indicates that the issue transcends immunology and is firmly inserted into debates on social determinants, institutional trust, and health communication.

Importance of Digital Communication and Online Environment Journals

The presence of journals specialized in digital communication is particularly relevant:

- Journal of Medical Internet Research (50 articles; 1949 citations).
- JMIR Infodemiology (21 articles; 263 citations).

These journals have become key spaces for research focused on: disinformation and misinformation, social network analysis, infodemic, text mining, NLP, and big data, digital behavior in response to vaccination campaigns.

The volume and citation levels demonstrate that studies on digital environments and social perception constitute one of the theoretical and methodological pillars of the field.

Convergence between Productivity and Impact

When jointly comparing “Quantity” and “Citations,” significant patterns emerge:

- Vaccines presents the highest productivity and one of the highest citation levels, positioning itself as the field’s reference journal.
- Human Vaccines and Immunotherapeutics, despite having fewer articles, surpasses several higher-volume journals in citations, suggesting high quality and influence of its publications.
- Journal of Medical Internet Research maintains a notable balance between productivity and citations, confirming the importance of studies on digital disinformation and public perception.
- Journals such as BMJ Open and IJERPH demonstrate the capacity to attract studies with broad perspectives: epidemiological, social, environmental, and behavioral.

General Interpretation

Table 1 reveals that scientific knowledge on perception, trust, and vaccine reluctance is primarily structured around three domains:

1. Vaccinology and immunization—Focused on biomedical factors, trust in efficacy, and analysis of vaccination programs.
2. Public health—Centered on inequalities, social determinants, community strategies, and health policies.
3. Digital communication and disinformation—Oriented toward the study of the impact of social networks, fake news, infodemics, and computational models.

This convergence confirms that it is a hybrid field where biomedical sciences, social sciences, and computational sciences interact, which explains the breadth and complexity of current scientific debates.

### 3.3. International Scientific Collaboration Networks

**Figure 2:**
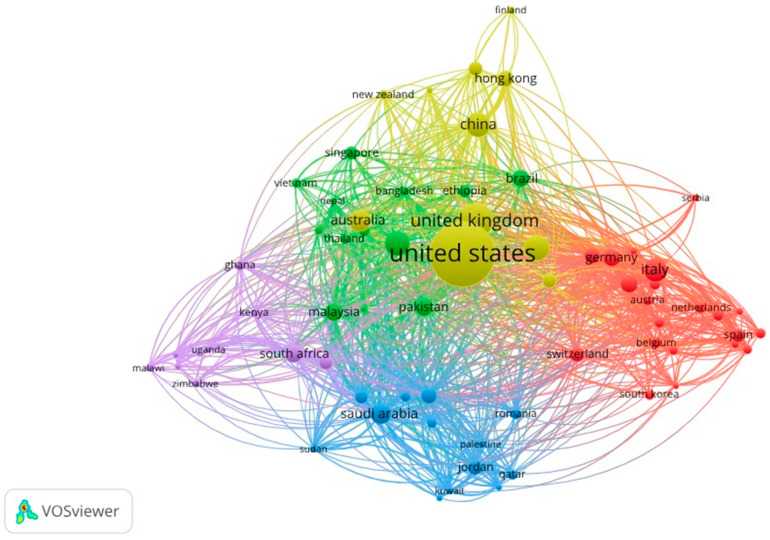
International Scientific Collaboration Network among Countries in Studies on Social Perception, Trust, and Vaccine Hesitancy.

Figure 2 represents the global structure of scientific collaboration between countries, visualized using VOSviewer and supported by the bibliometric table of documents, citations, and total link strength (TLS). The map reveals a densely interconnected network composed of several regional clusters and a reduced set of countries that act as articulating nodes of the international scientific system.

#### United States and United Kingdom as Central Axes of the Network

The countries with the largest node size and centrality are as follows:

- United States (571 documents; 330 citations; TLS = 422);
- United Kingdom (168 documents; 139 citations; TLS = 287).

These two countries form the hegemonic core of global collaboration. On the map, they are located in the central zone and exhibit an extraordinarily high number of connections with the majority of countries across all clusters. Their dominant position not only reflects productivity but also a structural capacity to coordinate, fund, and lead multinational research on vaccine hesitancy, digital disinformation, and public health policies.

Blue Cluster: East Asia and Scientific-Digital Powers

Includes China, Hong Kong, Finland, and Japan, among others:

- China (83 documents; 3213 citations; TLS = 113) stands out as the Asian country with the highest production and one of those reporting the greatest impact. Its research lines are associated with modeling public perception, social network analysis, and big data approaches to study disinformation and attitudes toward vaccination.
- The presence of Hong Kong, Japan, and Finland reflects a strong orientation toward computational methods and digital communication.

This area of the map shows considerable density of internal links, indicating intraregional cooperation, although always anchored in collaborations with the United States and United Kingdom.

Green Cluster: Asia-Pacific Region and Emerging Countries in Social Vaccinology

This cluster groups countries such as the following:

- India (83 documents; 56 citations; TLS = 140);
- Australia (62 documents; 145 citations; TLS = 134);
- Malaysia (45 documents; 110 citations; TLS = 116);
- Pakistan (43 documents; 1335 citations; TLS = 148);
- Indonesia, Bangladesh, Ethiopia, Vietnam, and Nepal.

India and Australia emerge as semi-peripheral nodes of high connectivity. In particular, India presents one of the highest levels of total link strength (TLS = 140), confirming its role as a regional articulator and its growth in health sciences and vaccine perception studies.

This cluster is one of the most diverse in geographic terms and reflects the expansion of research in middle-income countries, especially those related to vaccine acceptance, health literacy, and sociocultural analysis.

Red Cluster: Western Europe—Methodological and Epidemiological Core

Countries such as the following:

- Italy (74 documents; 243 citations; TLS = 82);
- Germany (43 documents; 716 citations; TLS = 134);
- France (33 documents; 173 citations; TLS = 110);
- Netherlands (17 documents; 107 citations; TLS = 53);
- Spain (25 documents; 1563 citations; TLS = 48);
- Belgium, Austria, Switzerland, and Portugal.

This cluster is one of the most cohesive and presents intense linkages between European countries. The high citation levels of countries such as Austria (1663 citations), Spain (1563), and Germany indicate the presence of highly influential studies, particularly systematic reviews, analyses of public policies, and comparative studies on vaccine hesitancy in the European Union.

Its orientation centers on epidemiology, health sociology, and transnational studies on risk communication.

Yellow-Violet Cluster: Middle East and North Africa

This cluster includes the following countries:

- Saudi Arabia (59 documents; 173 citations; TLS = 158);
- Egypt (23 documents; 604 citations; TLS = 119);
- United Arab Emirates (35 documents; 630 citations; TLS = 134);
- Qatar, Jordan, Iraq, Iran, and Lebanon.

Saudi Arabia stands out with a high TLS (158), demonstrating its active integration into multinational projects, especially in cooperation with the U.S. and United Kingdom.

This cluster is highly related to studies on institutional trust, religious factors, sociocultural barriers, and vaccine acceptance in multicultural contexts.

Purple Cluster: Sub-Saharan Africa

This cluster includes the following countries:

- South Africa (45 documents; 350 citations; TLS = 97);
- Ghana (13 documents; 1218 citations; TLS = 52);
- Kenya, Uganda, and Malawi.

In this group, low production is observed, but some countries present highly cited works, such as Ghana (1218 citations) and Malawi (1437 citations), indicating that certain local research has a great global impact, especially that related to infectious diseases, community trust, and childhood vaccination programs.

The density of links is lower, although connections with the United Kingdom are particularly strong due to historical collaborations and international funding.

Latin American Countries: Limited Presence in the Network

Although Latin America is the focus of the study, its representation in the network is minimal. From the countries in the table, only the following appear:

- Brazil (37 documents; 1101 citations; TLS = 63);
- Mexico (5 documents; TLS = 20, citations 307).

Brazil is the only Latin American country with a clear presence in the global network. Its TLS = 63 indicates active participation, but dependent on collaborations with the U.S., United Kingdom, and Europe. Mexico, although with low production, shows moderate impact in citations.

The absence of the majority of Latin American countries reflects a structural gap in international scientific participation, as well as a future opportunity to strengthen collaborative networks.

General Interpretation of the Map

Figure 2 evidences that the field presents high centralization, structured around two dominant poles—the United States and United Kingdom—that concentrate most of the production and act as the main distributors of scientific knowledge. Around this core, regional clusters with differentiated functions articulate, among which stand out East Asia, oriented toward technological approaches; Western Europe, characterized by epidemiological and methodological perspectives; the Middle East, where sociocultural dimensions prevail; Africa, centered on community trust and vaccination programs; and the Asia-Pacific, with a marked emphasis on vaccine acceptance and digital health. Despite being a key region for the study of vaccine hesitancy, Latin America shows marginal participation, revealing weak insertion into international collaboration networks. Overall, the network exhibits a multinuclear structure but strongly dependent on the Anglo-Saxon axis, which conditions the circulation of knowledge and limits the global visibility of research produced from peripheral regions.

### 3.4. Thematic Structure of the Scientific Field

**Figure 3:**
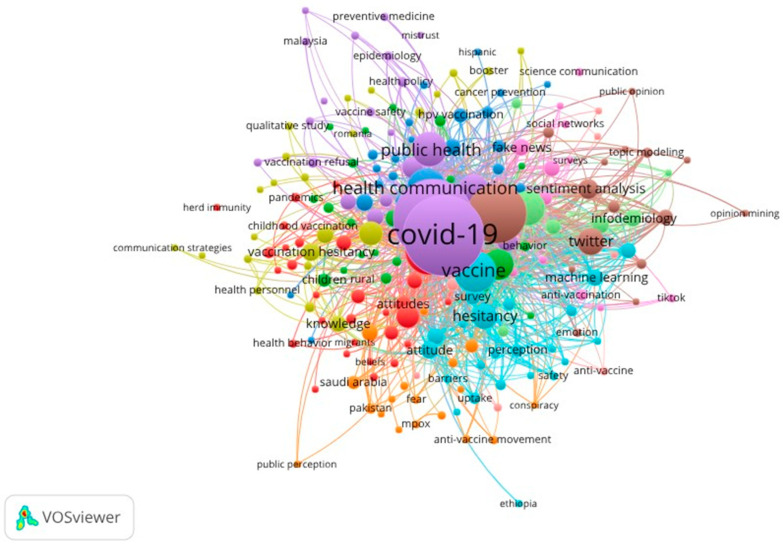
Keyword Co-occurrence Network: Thematic Structure in Studies on Social Perception, Trust, and Vaccine Hesitancy.

Figure 3 reveals a broad and highly interconnected thematic structure, in which keywords are organized around conceptual cores that articulate the scientific production on social perception, trust, and vaccine reluctance. The analysis of occurrences and total link strength (TLS) confirms the existence of well-defined clusters that reflect the interdisciplinary complexity of the field.

First, it is observed that COVID-19 constitutes the structuring axis of the thematic network. The keyword covid-19 presents the highest occurrence (673) and the highest TLS (2355), evidencing its central role in shaping the field. Its close linkage with coronavirus, sars-cov-2, pandemic, covid-19 vaccine, and covid-19 vaccines confirms that the pandemic acted as a catalyst that directed research toward the analysis of vaccine acceptance, risk communication, and the social response to the health crisis.

A second core is formed by the cluster of vaccine hesitancy and acceptance, which includes terms such as vaccine hesitancy (662 occurrences; TLS = 2168), hesitancy (63; 331), vaccine acceptance (105; 339), and vaccine confidence (45; 174). This group articulates with psychosocial constructs—attitudes, beliefs, risk perception, intention—all with high linkage levels, reflecting the importance of sociopsychological models for explaining the dynamics of trust, doubt, or rejection toward vaccines. The density of this cluster confirms that vaccine reluctance is understood as a multidimensional phenomenon traversed by cognitive, emotional, cultural, and contextual factors.

The third highlighted thematic domain corresponds to the cluster of communication, disinformation, and infodemic, one of the most robust in the network. Concepts such as health communication (152 occurrences; TLS = 499), social media (365; 1549), misinformation (127; 621), fake news (32; 115), infodemic (32; 190), and infodemiology (32; 243) show that the circulation of information—and particularly disinformation—constitutes a critical determinant of vaccination behavior. The strong presence of twitter (75; 464), sentiment analysis (43; 221), text mining, and machine learning reveals a marked trend toward computational methodologies to analyze digital discourses, social interactions, and conspiracy narratives related to vaccines.

Likewise, the network identifies a subfield centered on public health and epidemiology, where public health (124; 507), health promotion (23; 114), risk communication (11; 41), and epidemiology (9; 39) stand out. This cluster reflects interest in understanding the phenomenon from an institutional and population perspective, including topics such as pandemic preparedness, health policies, and community intervention strategies.

Another relevant component is the cluster associated with specific population groups, evidenced by terms such as children, adolescents, pregnancy, pregnant women, and healthcare workers. Their presence indicates the importance of understanding the determinants of vaccine acceptance in vulnerable or strategic groups for the implementation of immunization programs.

Finally, the network shows subclusters of specific vaccines—HPV vaccine, cervical cancer, influenza, childhood vaccination—that highlight consolidated research lines beyond COVID-19, especially in areas related to reproductive health and childhood vaccination.

Overall, Figure 3 evidences that the field is organized around a complex, multifocal, and highly interdisciplinary thematic structure, where epidemiological, sociopsychological, and communicational approaches converge. The centrality of COVID-19, the prominence of vaccine hesitancy, and the weight of digital disinformation reveal a scientific landscape where vaccine acceptance is understood as a social phenomenon deeply influenced by cognitive, cultural, and media factors. This thematic organization also expresses the maturity of the field and the methodological evolution toward mixed approaches and advanced analyses based on massive data and digital information mining.

### 3.5. Qualitative Analysis

Table 2 synthesizes the main characteristics of the most cited studies (*n* > 30) in the fields of social perception, trust, and vaccine hesitancy. This selection represents the most influential intellectual core within the investigated domain and allows for understanding how the most impactful scientific evidence evolves. For each study, the authors, year of publication, journal, number of citations, and a qualitative synthesis of the main findings obtained from their abstracts are detailed. The table constitutes a key input for the bibliometric-qualitative analysis, as it facilitates the identification of conceptual patterns, recurrent methodological approaches, and thematic trends that structure the contemporary debate on vaccine acceptance and rejection. Likewise, it enables contrasting the geographic, disciplinary, and theoretical diversity of the most influential contributions, providing a solid foundation for integrating bibliometric results with the qualitative interpretation of the literature.

The analysis of the fifty most cited studies in the sample reveals a highly consistent body of evidence surrounding the psychological, sociocultural, informational, and structural determinants that shape social perception, trust, and vaccine hesitancy. The included literature encompasses research published primarily between 2019 and 2022, with a notable predominance of studies developed during the COVID-19 pandemic, which explains the thematic concentration around vaccine acceptance, digital disinformation, and psychosocial factors influencing vaccination decisions.

Institutional trust emerges as the most robust determinant identified by the majority of the analyzed studies. Works such as those by Sallam et al. [39], Ruiz and Bell [19], and Jennings et al. [20] show that vaccination intention is strongly associated with the credibility attributed to governments, health systems, scientific authorities, and media outlets. The erosion of this trust is recurrently linked to doubts about governmental transparency, previous negative experiences with public institutions, and perceptions of inadequate pandemic management. In countries such as Algeria, Egypt, Iran, and Saudi Arabia, studies like those by Lounis et al. [33], Ogunleye et al. [8], and Khankeh et al. [9] reinforce that institutional trust is a decisive predictor in contexts where political tensions and structural inequality generate climates of social uncertainty.

Digital disinformation constitutes another transversal axis. Studies such as those by Piedrahita-Valdés et al. [24], Jamison et al. [31], and Baines et al. [32] provide compelling evidence that digital platforms amplify anti-vaccine narratives through echo chambers, emotionally charged content, and algorithmic microsegmentation. The analysis of advertising on Facebook [31] and interactions on Parler [32] shows that anti-vaccine messages appeal to political and moral identities, producing a discursive environment that contributes to social polarization and the delegitimization of health authorities. Similarly, studies employing text mining and machine learning demonstrate that negative or conspiratorial content has higher diffusion rates than institutional messages.

Regarding risk perception, the findings of Olson et al. [21], Montalti et al. [25], Zhang et al. [18], and Ashkenazi et al. [36] indicate that the subjective valuation of personal and family risk directly influences vaccine acceptance. Individuals who perceive themselves as vulnerable or consider the disease’s severity to be high show greater intention to vaccinate. Conversely, risk minimization, perceptions of invulnerability, and beliefs in natural immunity are associated with higher levels of hesitancy, particularly among young people and healthy adults. In the case of pregnant women, Zhang et al. [18] show that uncertainty about potential effects on the fetus constitutes a critical barrier to accepting vaccination.

The literature also highlights the role of health professionals as essential actors in mediating trust. However, studies such as those by Riad et al. [28] (both in dental students and university students) evidence that even among future professionals, there are significant levels of vaccine hesitancy associated with fears of adverse effects, insufficient training in immunology, or exposure to contradictory information. Despite this, the recommendation from a professional continues to be one of the most potent strategies for increasing acceptance, as documented by Purvis et al. [29] and Evans and French [34], who emphasize the importance of empathetic and personalized communication.

The analysis of the sociocultural dimension shows that hesitancy cannot be understood without considering the local context. In studies conducted in Nigeria, South Africa, Jordan, Malaysia, and India—such as those by Wiyeh et al. [35], Purvis et al. [29], Romate et al. [44], and Umakanthan et al. [27]—it is observed that factors such as religion, family dynamics, educational capital, and socioeconomic inequalities influence vaccination decision-making. In many cases, the authors recommend developing culturally adapted strategies that involve community leaders and utilize legitimate communication channels within each culture.

The evidence also underscores the influence of public communication, particularly the clarity, coherence, and consistency of messages. Studies such as that by Rzymski et al. [26] show that the mere transmission of scientific evidence is insufficient; messages must be comprehensible, emotionally sensitive, and culturally appropriate. The literature agrees that contradictory messages, lack of clarity in health guidelines, and frequent variations in public policies generate confusion, reduce institutional credibility, and increase hesitancy.

From a methodological standpoint, the fifty studies share a predominant use of cross-sectional designs, population surveys, content analysis, text mining, regression models, and mixed approaches. Research such as that by Bradshaw et al. [5] highlights the value of combining digital methods with traditional qualitative analyses to better understand the dynamics of disinformation and conspiracy beliefs.

Finally, the reviewed literature identifies five robust thematic cores:

1. Institutional trust as the central axis of vaccine acceptance [19,33,39].
2. Digital disinformation, social networks, and anti-vaccine discourses [24,31].
3. Risk perception and emotions linked to decision-making [21,25].
4. Vulnerable groups and specific populations [18,28].
5. Institutional communication strategies and their effectiveness [26,34]).

Overall, this corpus evidences that vaccine hesitancy is a multidimensional phenomenon in which individual, cognitive, sociocultural, emotional, and structural factors articulate. The fifty analyzed studies provide a solid theoretical and empirical foundation for developing integral communication strategies, strengthening health literacy, and designing evidence-based immunization policies.

## 4. Discussion

The results of this study, which combine a comprehensive bibliometric analysis with a qualitative content synthesis of the most cited articles, allow for understanding the evolution, concentration, approaches, and research gaps on social perception, trust, and vaccine hesitancy. The identified patterns show that the field has grown rapidly since 2020, driven primarily by the COVID-19 pandemic, which aligns with reports from highly cited studies such as those by Martin et al. [1], who emphasize the decisive role of global uncertainty and the amplification of perceived risk in vaccination behavior.

From a structural perspective, the bibliometric results show a high geographic centralization, where the United States and the United Kingdom constitute the hegemonic poles of scientific production. This concentration is consistent with the findings of Allington et al. [4] and Guess et al. [7], who analyze how research infrastructure, data access, and the influence of academic powers condition the development of narratives on vaccines and disinformation. However, this phenomenon entails a significant bias: the dominant literature predominantly reflects Anglo-Saxon explanatory frameworks, while key regions for studying hesitancy—such as Latin America, Africa, and the Middle East—remain underrepresented, despite contributing relevant evidence in research such as that by Ogunleye et al. [8], Khankeh et al. [9], and Al-Mulla et al. [15].

At the thematic level, keyword co-occurrence reveals three conceptual axes that articulate the field:

1. Disinformation and anti-vaccine narratives, strongly analyzed in works such as Bradshaw et al. [5] and Wawrzuta et al. [12], where emotionality, conspiracy, and digital virality appear as central determinants of hesitancy.
2. Institutional trust and health communication, developed in studies such as Dempsey et al. [10], Middleman et al. [11], and Leader et al. [49], who show that medical recommendations, message clarity, and risk transparency are stronger predictors than factual knowledge.
3. Psychobehavioral determinants, analyzed by Moscardino et al. [2], Meyer et al. [14], and Han et al. [13], where variables such as fear, anxiety, perceived susceptibility, and prior memories influence vaccination intention.

The qualitative findings allow for deepening this reading. For example, while the bibliometric analysis reveals that the United States leads in production and citation, qualitative studies from that country—such as Allington et al. [4] and Guess et al. [7]—evidence a major internal challenge: highly polarized disinformation and the influence of digital echo chambers. In contrast, research from Asia—such as Zhang et al. [18] and Han et al. [13]—shows scenarios where governmental trust positively modulates vaccination intention, suggesting structural differences between sociopolitical systems that affect citizen perception.

Likewise, studies from the Middle East and Africa, such as Al-Mulla et al. [15] and Ogunleye et al. [8], allow for observation of how cultural, religious, and community factors acquire distinct weight in contexts where formal institutions do not play a predominant role. This contrasts with European literature, such as Frascella et al. [17] and Mascherini and Nivakoski [43], which centers its analysis on socioeconomic inequities, state trust, and evidence-based public policies.

Overall, the results show that vaccine hesitancy is not a uniform phenomenon, but multidimensional, contextual, and dynamic, traversed by the following:

- individual psychological characteristics,
- interpersonal and institutional trust structures,
- circulation of information and disinformation,
- sociocultural conditions,
- and structural inequalities.

These elements converge with the bibliometric finding of a multinuclear network, but dependent on the Anglo-Saxon axis, a phenomenon that conditions the global narrative on vaccine hesitancy.

### 4.1. Practical Implications

The implications of these findings are relevant for the formulation of public health policies and strategies.

First, the most cited studies confirm that institutional trust is the most robust determinant of vaccine acceptance. Research such as that by Dempsey et al. [10] and Middleman et al. [11] shows that direct professional recommendations have a greater impact than any mass media campaign. Therefore, health systems must strengthen interpersonal communication channels and train professionals to address doubts from empathetic, culturally sensitive, and evidence-based perspectives.

Second, the bibliometric and qualitative analysis converges on the fact that disinformation operates as one of the main inhibitors of vaccination intention. Studies such as those by Bradshaw et al. [5], Allington et al. [4], and Guess et al. [7] explain how anti-vaccine narratives appeal to emotions, existential fears, and conspiracy theories, managing to impact even individuals with good scientific literacy. This demands the creation of digital surveillance systems (social listening) that respond early to rumors and misleading content.

Third, the reviewed studies show that hesitancy is deeply traversed by cultural and community factors, especially in Global South regions, as evidenced by Ogunleye et al. [8] and Khankeh et al. [9]. Strategies must adapt to local realities, incorporating community leaders, cultural narratives, and accessible communication.

Finally, studies such as Sallam et al. [6] and Riad et al. [37] evidence that even informed populations, such as health workers, can exhibit hesitancy when uncertainty about safety predominates; therefore, risk communication must be clear, verifiable, and based on comparative evidence.

### 4.2. Future Research Areas

The cross-referencing of bibliometric results with qualitative analysis allows for identifying at least five priority lines of research.

1. Intercultural models of vaccine hesitancy. The dominant literature reflects the Global North. Studies integrating Latin American and African contexts are needed, where cultural and structural variables differ widely. Works such as those by Ogunleye et al. [8] show the potential of these approaches.
2. Longitudinal and evolutionary studies. Most included studies are cross-sectional (e.g., [13,40]). There is a need to understand how hesitancy changes over time, especially post-COVID-19.
3. Intersection between artificial intelligence, algorithms, and disinformation. Computational analysis studies (e.g., [5,18]) show the need to deepen the role of digital platforms in amplifying risk.
4. Vulnerable populations and minorities. Studies such as Moscardino et al. [2] evidence disparities, but there is a scarcity of research on rural communities, migrants, indigenous groups, and low-income populations.
5. Ethics and governance of health communication. The use of AI, algorithmic moderation, and persuasive strategies requires rigorous ethical analysis.

In addition to the core themes identified through the bibliometric and qualitative content synthesis, the reviewed literature also highlights other relevant determinants of vaccine hesitancy that merit consideration. Several studies report the emergence of vaccine fatigue, particularly in the context of repeated booster campaigns, where sustained exposure to vaccination messaging may reduce motivation and increase ambivalence toward continued uptake. Moreover, access-related barriers—including logistical constraints, availability, and structural inequalities—are frequently associated with higher levels of hesitancy, especially in low-resource or marginalized settings.

The literature also underscores the role of perceived and experienced side effects as a critical factor shaping vaccination decisions, with concerns about adverse reactions contributing to uncertainty and refusal in both general populations and healthcare workers. In addition, vaccine-specific characteristics influence acceptance, as newly developed vaccines are often met with higher levels of skepticism due to perceived uncertainty regarding long-term safety and effectiveness. Although these factors did not emerge as dominant clusters in the bibliometric mapping, they are consistently reported across influential studies and should be considered complementary dimensions in the interpretation of vaccine hesitancy.

### 4.3. Limitations

This study has certain limitations that should be considered when interpreting the findings. The analysis is constrained by the structure of the international scientific literature indexed in Scopus and Web of Science, which is characterized by a pronounced geographic imbalance. As a result, the bibliometric dataset disproportionately reflects research produced in and focused on Global North contexts, while contributions from Latin America and other underrepresented regions remain comparatively limited. This geographic asymmetry represents a structural characteristic of the field that shapes the visibility of research agendas, dominant narratives, and methodological approaches.

Accordingly, although this study examines vaccine hesitancy, trust, and social perception as globally debated phenomena, the findings should be interpreted as an analysis of dominant frameworks within the international literature rather than as a geographically representative account. The limited presence of regionally grounded empirical studies from Latin America restricts the extent to which context-specific dynamics can be captured through bibliometric patterns alone.

In addition, the qualitative content synthesis was based on a citation threshold (*n* > 30) to identify influential publications, which may further privilege highly cited studies and reinforce the prominence of globally visible research. While this criterion was applied to examine conceptual influence rather than regional representativeness, it introduces an additional bias toward well-established scholarly outputs.

Finally, this study relies on indexed peer-reviewed literature and does not incorporate gray literature, policy documents, or locally disseminated research, which may contain relevant insights into vaccine hesitancy in specific sociocultural contexts. Future research would benefit from complementary approaches that foreground regionally situated empirical studies, alternative data sources, and methodologies explicitly designed to amplify underrepresented perspectives.

## 5. Conclusions

The present study achieved its objective of analyzing in an integrated manner social perception, trust, and vaccine hesitancy through a bibliometric and qualitative approach applied to the 48 most cited studies in contemporary scientific literature. The results allow establishing that vaccine hesitancy is a global, complex phenomenon deeply influenced by sociocultural, communicational, and psychobehavioral factors that interact dynamically in different geographic contexts.

From the bibliometric perspective, a marked geographic concentration of knowledge was identified, dominated by the United States and United Kingdom, whose collaboration, production, and citation networks configure the central structure of the field. This hegemony conditions the circulation of knowledge and limits the visibility of studies from key regions for understanding vaccine hesitancy, such as Latin America, Africa, and the Middle East, although these contribute valuable perspectives on structural inequalities, cultural barriers, and institutional distrust. Leading journals—Vaccines, Vaccine, Human Vaccines and Immunotherapeutics, JMIR—reflect an orientation toward multidisciplinary approaches that integrate public health, epidemiology, communication, and digital social sciences.

The network analysis showed the coexistence of solid thematic clusters: digital disinformation, health communication, risk psychology, institutional trust, and vaccine safety. These findings were confirmed and expanded by the qualitative analysis, which evidences that vaccine hesitancy does not respond to cognitive deficits, but to social trust structures, historical experiences, emotions, and cultural frameworks. Studies by Martin et al. [1], Dempsey et al. [10], Allington et al. [4], Bradshaw et al. [5], Han et al. [13], among others, show consistent patterns: professional recommendation is the strongest predictor of acceptance, while disinformation—particularly that circulating on social networks—erodes perceived safety and strengthens anti-vaccine narratives.

The cross-referencing between bibliometric and qualitative results reveals three central conclusions. First, vaccine hesitancy must be understood as a contextual and relational phenomenon, determined by the interaction between institutional trust, source credibility, and prior experiences with health systems. Second, digital dynamics have modified the way individuals interpret risk, generating saturated informational environments where emotion surpasses evidence. Third, cultural and socioeconomic determinants emerge as critical factors in underrepresented regions, evidencing the need to expand the geographic and epistemological scope of research.

Finally, this study demonstrates that the combined analysis of scientific production metrics and qualitative synthesis allows for a more complete understanding of vaccine hesitancy, overcoming reductionist approaches focused solely on individual behaviors. It is concluded that strengthening trust, improving public communication, and developing culturally adapted strategies are urgent priorities for health systems. Likewise, the need for comparative, interdisciplinary, and intercultural research is highlighted to advance toward more inclusive and representative explanatory models of global diversity.

Overall, the findings confirm that vaccine hesitancy is not an information problem, but one of trust, legitimacy, and social structure, and that its approach requires integral approaches sustained by scientific evidence and cultural sensitivity.

## Figures and Tables

**Table 1:** Top Journals by Number of Publications and Citations in Research on Social Perception, Trust, and Vaccine Hesitancy.

Source Title	Documents	Citations
Vaccines	135	3969
Vaccine	103	2236
Human Vaccines and Immunotherapeutics	57	2742
Journal of Medical Internet Research	50	1949
PLOS ONE	49	1568
BMC Public Health	40	1092
Frontiers in Public Health	40	570
International Journal of Environmental Research and Public Health	37	1266
BMJ Open	26	665
JMIR Infodemiology	21	263

**Table 2:** Most Cited Studies on Social Perception, Trust, and Vaccine Hesitancy: Key Characteristics and Findings.

#	Authors	Citations	Title	Main Findings
1	Sallam et al. [6]	521	High rates of COVID-19 vaccine hesitancy and its association with conspiracy beliefs: A study in Jordan and Kuwait among other Arab countries	High levels of vaccine hesitancy associated with conspiracy narratives, institutional distrust, and distorted risk perceptions.
2	Ruiz and Bell [19]	512	Predictors of intention to vaccinate against COVID-19: Results of a nationwide survey	Vaccination intention is explained by trust in science, perceived severity, and risk-benefit assessment; disinformation predicts refusal.
3	Jennings et al. [20]	446	Lack of trust, conspiracy beliefs, and social media use predict COVID-19 vaccine hesitancy	Combination of low trust, conspiracy beliefs, and heavy social media use strongly predicts vaccine hesitancy.
4	Olson et al. [21]	163	Addressing parental vaccine hesitancy towards childhood vaccines in the united states: A systematic literature review of communication interventions and strategies	Parental hesitancy arises from fear of adverse effects, vaccine overload concerns, and weak empathetic communication.
5	Shakeel et al. [22]	137	Global COVID-19 Vaccine Acceptance: A Systematic Review of Associated Social and Behavioral Factors	Geographic variability in acceptance; trust, efficacy perception, and digital disinformation shape attitudes.
6	Al-Sanafi and Sallam [23]	121	Psychological determinants of COVID-19 vaccine acceptance among healthcare workers in Kuwait: A cross-sectional study using the 5c and vaccine conspiracy beliefs scales	Anxiety, fear, and perceived susceptibility influence vaccination intention; emotional factors require targeted interventions.
7	Piedrahita-Valdés et al. [24]	117	Vaccine hesitancy on social media: Sentiment analysis from June 2011 to April 2019	Polarization and increasing anti-vaccine content on social media shape public opinion via conspiratorial rhetoric.
8	Montalti et al. [25]	116	Would parents get their children vaccinated against SARS-CoV-2? Rate and predictors of vaccine hesitancy according to a survey over 5000 families from bologna, Italy	Perceived risk and trust in safety predict acceptance; concerns arise from rapid development and pediatric adverse effects.
9	Rzymski et al. [26]	111	The strategies to support the COVID-19 vaccination with evidence-based communication and tackling misinformation	Effective communication must combine evidence-based messages with moderate emotional appeals and cultural sensitivity.
10	Umakanthan et al. [27]	106	COVID-19 Vaccine Hesitancy and Resistance in India Explored through a Population-Based Longitudinal Survey	Identified sociocultural barriers, misinformation, and inequalities influencing hesitancy (despite retraction).
11	Riad et al. [28]	92	Global prevalence and drivers of dental students’ COVID-19 vaccine hesitancy	Hesitancy linked to low perceived susceptibility and safety concerns among future healthcare professionals.
12	Purvis et al. [29]	76	Trusted sources of COVID-19 vaccine information among hesitant adopters in the united states	Interpersonal communication with health professionals and community leaders outweighs mass campaigns.
13	Riad et al. [30]	75	Prevalence and drivers of COVID-19 vaccine hesitancy among Czech university students: National cross-sectional study	Hesitancy tied to institutional distrust and safety concerns; recommends youth-targeted interventions.
14	Jamison et al. [31]	72	Vaccine-related advertising in the Facebook Ad Archive	Anti-vaccine groups use microtargeted advertising to shape public perceptions.
15	Baines et al. [32]	69	#Scamdemic, #Plandemic, or #Scaredemic: What Parler Social Media Platform Tells Us about COVID-19 Vaccine	Parler hosts ideologically homogeneous anti-vaccine communities amplifying conspiracy narratives.
16	Lounis et al. [33]	68	COVID-19 Vaccine Booster Hesitancy (VBH) and Its Drivers in Algeria: National Cross-Sectional Survey-Based Study	Safety concerns, unclear official information, and distrust hinder booster acceptance.
17	Evans and French [34]	65	Demand creation for COVID-19 vaccination: Overcoming vaccine hesitancy through social marketing	Emotionally sensitive and segmented messages effectively increase acceptance.
18	Wiyeh et al. [35]	62	Social media and HPV vaccination: Unsolicited public comments on a Facebook post by the Western Cape Department of Health provide insights into determinants of vaccine hesitancy in South Africa	Digital disinformation and emotional content shape vaccine attitudes; recommends continuous monitoring.
19	Ashkenazi et al. [36]	62	The relationship between parental source of information and knowledge about measles/measles vaccine and vaccine hesitancy	Trust in pediatricians predicts acceptance; unverified media exposure increases refusal.
20	Martin et al. [1]	55	“Vaccines for pregnant women…?! Absurd”—Mapping maternal vaccination discourse and stance on social media over six months	Institutional trust and message credibility shape public perception; hesitancy is multidimensional.
21	Dempsey et al. [10]	53	Parent report of provider HPV vaccine communication strategies used during a randomized, controlled trial of a provider communication intervention	Clear doctor recommendations increase acceptance; conflicting information reduces intention.
22	Allington et al. [4]	53	Media usage predicts intention to be vaccinated against SARS-CoV-2 in the US and the UK	Conspiratorial content reduces trust; misinformation outweighs scientific evidence.
23	Wawrzuta et al. [12]	51	What Arguments against COVID-19 Vaccines Run on Facebook in Poland: Content Analysis of Comments	Anti-vaccine content uses emotional narratives, increasing reach; calls for stronger persuasive communication.
24	Riad et al. [37]	50	Monkeypox Knowledge and Vaccine Hesitancy of Czech Healthcare Workers: A Health Belief Model (HBM)-Based Study	Trust in efficacy and perceived susceptibility predict acceptance among healthcare workers.
25	Küçükali et al. [16]	50	Vaccine Hesitancy and Anti-Vaccination Attitudes during the Start of COVID-19 Vaccination Program: A Content Analysis on Twitter Data	Safety concerns dominate hesitancy; transparency in risk communication increases acceptance.
26	Meyer et al. [14]	49	Vaccine hesitancy and Web 2.0: Exploring how attitudes and beliefs about influenza vaccination are exchanged in online threaded user comments	Negative attitudes shaped by local narratives; community involvement improves trust.
27	Hatmal et al. [38]	49	Reported Adverse Effects and Attitudes among Arab Populations Following COVID-19 Vaccination: A Large-Scale Multinational Study Implementing Machine Learning Tools in Predicting Post-Vaccination Adverse Effects Based on Predisposing Factors	Sociodemographic factors and misinformation shape hesitancy; trust in science protects against disinformation.
28	Sallam et al. [39]	49	Attitude towards HPV Vaccination and the Intention to Get Vaccinated among Female University Students in Health Schools in Jordan	Disinformation during the pandemic reduces acceptance; recommends integrated communication strategies.
29	Middleman et al. [11]	48	Vaccine Hesitancy in the Time of COVID-19: Attitudes and Intentions of Teens and Parents Regarding the COVID-19 Vaccine	Healthcare personnel recommendations most strongly predict acceptance; family trust is key.
30	Pedersen et al. [40]	48	Strategic health communication on social media: Insights from a Danish social media campaign to address HPV vaccination hesitancy	Parents’ decisions shaped by safety perceptions and message clarity; personalized communication needed.
31	Han et al. [13]	46	Confidence, Acceptance and Willingness to Pay for the COVID-19 Vaccine among Migrants in Shanghai, China: A Cross-Sectional Study	Trust in authorities and perceived efficacy predict acceptance; consistent messaging reduces hesitancy.
32	Loft et al. [41]	45	Using Facebook to increase coverage of HPV vaccination among Danish girls: An assessment of a Danish social media campaign	Parental doubts influenced by media contradictions and lack of guidance; empathetic communication required.
33	Al-Mulla et al. [15]	44	COVID-19 Vaccine Hesitancy in a Representative Education Sector Population in Qatar	Religiosity, trust, and severity perception shape intention; culturally sensitive strategies needed.
34	Barry et al. [42]	43	COVID-19 vaccine uptake among healthcare workers in the fourth country to authorize BNT162b2 during the first month of rollout	Concerns about effects persist; trust in government improves acceptance, rumors reduce it.
35	Guess et al. [7]	43	The sources and correlates of exposure to vaccine-related (mis)information online	Brief exposure to false content measurably decreases intention; recommends media literacy.
36	Frascella et al. [17]	42	Effectiveness of email-based reminders to increase vaccine uptake: a systematic review	Acceptance mediated by perceived benefits vs. fears; recommends population-specific messaging.
37	Mascherini and Nivakoski [43]	42	COVID-19 vaccine uptake among healthcare workers in the fourth country to authorize BNT162b2 during the first month of rollout	Socioeconomic inequality and disinformation shape attitudes; vulnerable groups require tailored interventions.
38	Romate et al. [44]	41	What Contributes to COVID-19 Vaccine Hesitancy? A Systematic Review of the Psychological Factors Associated with COVID-19 Vaccine Hesitancy	Hesitancy linked to structural barriers, negative experiences, and unclear information.
39	Moscardino et al. [2]	41	Sociodemographic and psychological correlates of COVID-19 vaccine hesitancy and resistance in the young adult population in Italy	Anxiety and health uncertainty reduce intention; family support increases acceptance.
40	Novilla et al. [45]	38	Why Parents Say No to Having Their Children Vaccinated against Measles: A Systematic Review of the Social Determinants of Parental Perceptions on MMR Vaccine Hesitancy	Trust depends on narrative quality and transparency; community participation strengthens adherence.
41	Citu et al. [46]	38	Determinants of COVID-19 Vaccination Hesitancy among Romanian Pregnant Women	Risk perception and clinical experience predict acceptance; hesitancy linked to information fatigue.
42	Sundstrom et al. [47]	38	Correcting HPV Vaccination Misinformation Online: Evaluating the HPV Vaccination NOW Social Media Campaign	Combining evidence with narratives increases impact; trust and empathy essential.
43	Zhao et al. [48]	37	Public Willingness and Determinants of COVID-19 Vaccination at the Initial Stage of Mass Vaccination in China	Fear of contagion increases acceptance; social distrust reduces willingness.
44	Bradshaw et al. [5]	34	Propagandizing anti-vaccination: Analysis of Vaccines Revealed documentary series	Anti-vaccine communities use emotional victimization narratives; strategic monitoring required.
45	Zhang et al. [18]	33	Vaccine Resistance and Hesitancy among Older Adults Who Live Alone or Only with an Older Partner in Community in the Early Stage of the Fifth Wave of COVID-19 in Hong Kong	Rumor spread accelerates due to lack of verification; official presence in networks reduces distrust.
46	Ogunleye et al. [8]	32	Coronavirus Disease 2019 (COVID-19) Pandemic across Africa: Current Status of Vaccinations and Implications for the Future	Unequal access to credible information amplifies hesitancy; recommends leader-based interventions.
47	Khankeh et al. [9]	32	The Barriers, Challenges, and Strategies of COVID-19 (SARS-CoV-2) Vaccine Acceptance: A Concurrent Mixed-Method Study in Tehran City, Iran	Safety concerns, adverse effects, and distrust shape refusal; clear information essential.
48	Leader et al. [49]	32	Understanding the messages and motivation of vaccine hesitant or refusing social media influencers	Targeted, segmented campaigns are more effective; credibility of messengers matters.

## Data Availability

The data supporting the findings of this study are derived from publicly available bibliographic databases (Scopus and Web of Science). Processed datasets generated during the analysis are available from the corresponding authors upon reasonable request.
